# Treatable Neonatal Molybdenum Cofactor Deficiency: Rapid Demise Despite Rapid Biochemical Diagnosis

**DOI:** 10.1002/jmd2.70061

**Published:** 2026-01-11

**Authors:** Molly M. Crenshaw, Yasmeen Midgette, Shruthi Mohan, Ruhan Wei, Mariele Anneling, Monika Williams, Milap Patel, Benjamin T. Cocanougher, Sarah P. Young, Dmitriy Niyazov, Areeg El‐Gharbawy, Ashlee R. Stiles

**Affiliations:** ^1^ Division of Medical Genetics, Department of Pediatrics Duke University Medical Center Durham North Carolina USA; ^2^ Division of Neonatology, Department of Pediatrics Duke University Medical Center Durham North Carolina USA; ^3^ Department of Pathology, Molecular Diagnostics Laboratory Duke University Health System Durham North Carolina USA; ^4^ Department of Pathology Duke University Medical Center Durham North Carolina USA; ^5^ Duke University Health System Clinical Laboratories Durham North Carolina USA; ^6^ Biochemical Genetics Laboratory Duke University Health System Durham North Carolina USA

**Keywords:** fosdenopterin, molybdenum cofactor deficiency, newborn screening, *S*‐sulfocysteine

## Abstract

Molybdenum cofactor deficiency (MoCD) is an inborn error of metabolism included in the differential for refractory neonatal seizures. The prognosis is guarded, with a median reported age of death between 2.4 and 3.0 years. Mortality is primarily due to seizures and lower respiratory tract infections. MoCD has a distinct biochemical profile, characterized by elevated urinary *S*‐sulfocysteine, xanthine, and hypoxanthine, and low or undetectable serum and urine uric acid levels. A disease‐altering treatment is available for MoCD Type A; however, due to the rarity of the condition, its natural history remains poorly understood. We present a patient with neonatal‐onset refractory seizures, whose biochemical testing, performed within 24 h of specimen receipt in the laboratory, revealed a pattern consistent with MoCD. Before the genetics team could disclose the preliminary diagnosis, the patient demised, without evidence of worsening seizures or respiratory infection. Results of genome sequencing, guided by biochemical findings, identified double homozygous pathogenic variants in *MOCS1* (associated with MoCD Type A). Although the biochemical genetics laboratory's protocol for analyzing and reporting *S*‐sulfocysteine levels within 48 h enabled a rapid preliminary diagnosis, the patient's condition deteriorated too quickly to initiate disease‐altering treatment, progressing more rapidly than described in the literature. We discuss potential hypotheses for his rapid decline and the broader implications for the field of biochemical genetics.

## Take‐Home Message

As more disease‐altering treatments emerge for inborn errors of metabolism, this case underscores the urgent need for robust natural history data, faster diagnostics, and more readily available treatments, especially at large academic medical centers.

## 1 Introduction

Molybdenum cofactor deficiency (MoCD) is a rare, autosomal recessive inborn error of metabolism (IEM) characterized by refractory seizures, severe encephalopathy, hypotonia, feeding difficulties, and acquired microcephaly [1]. Symptoms typically present in the neonatal or early infantile period [2]. Despite a 72%–75% survival rate beyond the first year of life, overall mortality in early childhood remains high, with a median age of death in untreated individuals between 2.4 and 3 years [2, 3, 4]. Death is most commonly attributed to seizures, lower respiratory infections, intracranial hemorrhage, and sepsis, though rare cases of rapid onset with progressive apnea and reduced consciousness have also been reported [3, 4].

The molybdenum cofactor is essential for the activity of four enzymes: sulfite oxidase, xanthine dehydrogenase, aldehyde oxidase, and mitochondrial amidoxime‐reducing component [1, 5, 6]. Four genes—*MOCS1, MOCS2, MOCS3*, and *GPHN*—encode the enzymes required for molybdenum cofactor biosynthesis [7]. In 2021, the US Food and Drug Administration approved fosdenopterin, a cyclic pyranopterin monophosphate (cPMP) and precursor to molybdenum [7], as the first disease‐modifying treatment for MoCD type A caused by *MOCS1* deficiency [8]. Clinical studies have shown that fosdenopterin is safe and effective when initiated early [7, 9], and current guidelines recommend starting treatment as soon as a biochemical suspicion of MoCD arises [7].

We present a case of MoCD type A in a neonate with rapid biochemical diagnosis based on elevated *S*‐sulfocysteine levels. Despite expedited protocols in place by the laboratory, the patient experienced an unusually rapid clinical decline. We explore potential explanations for this outcome and discuss the broader implications for the field of biochemical genetics.

## 2 Methods

Review of the patient's medical record and clinical parameters was performed by the clinical team. Informed consent with a witness was obtained from the father to share clinical findings of the proband and family.

Duke University Health System's (DUHS) CLIA/CAP‐certified Biochemical Genetics Laboratory performed urinary creatinine and *S*‐sulfocysteine analysis according to standard operating procedures. Creatinine analysis was performed by alkaline picrate (Jaffe reaction) method. *S*‐sulfocysteine was analyzed as a butyl ester by stable‐isotope dilution ultra‐performance liquid chromatography–tandem mass spectrometry (UPLC‐MS/MS) (unpublished method). Plasma uric acid analysis was performed by DUHS CLIA/CAP‐certified Central Automated Laboratory using a colorimetric method according to standard operating procedure. Urinary purines and pyrimidines analysis was performed by LC‐MS/MS by Mayo Medical Laboratories according to published protocols [10, 11].

Rapid trio genome sequencing using genomic DNA of the patient and both parents was performed by GeneDx.

## 3 Case Report

### 3.1 Clinical Presentation

The patient was a term male neonate, born at 40 weeks and 3 days of gestation, with a birth weight of 4167 g (symmetrically large for gestational age with birth weight, length, and head circumference all near the 90th percentile), to a gravida 3, para 3 mother. Both parents are of Nepalese descent, and there was no known history of consanguinity. Prenatal ultrasound revealed a prominent cisterna magna as the only abnormal finding. At delivery, the infant exhibited respiratory distress requiring continuous positive airway pressure. Apgar scores were 8 and 8 at 1 and 5 min, respectively. By Day of Life (DOL) 2, he was weaned to high‐flow nasal cannula and had initiated oral feeds. On DOL 3, the patient developed irritability, followed by the abrupt onset of refractory seizures on DOL 5. Seizure management included multiple doses of levetiracetam, phenobarbital, and fosphenytoin, with no significant clinical improvement. A trial of pyridoxine was also ineffective. Newborn metabolic screening was unremarkable and initial laboratory evaluations revealed normal anion gap, ammonia and lactate levels. Brain MRI demonstrated bilateral dysmorphic ventricles, cerebellar hypoplasia, a thin corpus callosum, and bilateral basal ganglia signal abnormalities, without evidence of hypoxic–ischemic injury. Physical examination was notable for a prominent philtrum, microstomia, and deep plantar creases. Family history was non‐contributory, with both parents and two older siblings (ages 4 and 7 years) reported as healthy.

### 3.2 Diagnostic Evaluation and Clinical Deterioration

On DOL 9, a day after transfer to our institution, the genetics team recommended trio rapid genome sequencing along with comprehensive in‐house biochemical testing to investigate a potential metabolic cause of the seizures. This included an acylcarnitine profile, free and total carnitine, plasma amino acids, urine organic acids, urine S‐sulfocysteine, and creatine and guanidinoacetate levels in both urine and plasma. On DOL 10, approximately 6 h after urine was received by the DUHS Biochemical Genetics Laboratory, a markedly elevated urine *S*‐sulfocysteine level of 1592 μmol/g creatinine (reference range: < 80) was reported to the ordering provider, with read‐back verification. In response, additional confirmatory testing was initiated, including in‐house plasma uric acid, plasma homocysteine and send‐out testing for urinary purine/pyrimidine analysis. Plasma uric acid was undetectable, strongly suggesting MoCD, and plasma homocysteine concentration was low. Urinary purine and pyrimidine analysis further supported the diagnosis of MoCD, confirming the previously observed elevated S‐sulfocysteine and low uric acid levels, and additionally revealing elevated xanthine and decreased hypoxanthine concentrations (Table 1). An echocardiogram on DOL 10 revealed mild septal flattening with otherwise normal biventricular size and systolic function.

**TABLE 1:** Summary of clinically relevant first‐tier and confirmatory laboratory findings.

	Result	Reference range	Interpretation
Diagnostic testing^a^			
Urine *S*‐sulfocysteine	1592 μmol/g Cr	< 80 μmol/g Cr	Markedly elevated
Confirmatory testing^a^			
Plasma uric acid	< 0.5 mg/dL	2.0–7.0 mg/dL	Undetectable
Urine *S*‐sulfocysteine^b^	380 μmol/g Cr	≤ 97 μmol/g Cr	Elevated
Urine xanthine^b^	149 mmol/mol Cr	≤ 54 mmol/mol Cr	Elevated
Urine uric acid^b^	32 mmol/mol Cr	350–2500 mmol/mol Cr	Markedly decreased
Urine hypoxanthine^b^	5 mmol/mol Cr	≤ 65 mmol/mol Cr	Normal
Additional laboratory testing^c^			
Arterial pH	7.1	7.35–7.45	Metabolic acidosis
pCO_2_	27 mmHg	60–76 mmHg	Respiratory compensation
Bicarbonate	8 mmol/L	17–26 mmol/L	Low
Base deficit	20 mmol/L	−3–3 mmol/L	Severe metabolic acidosis
Lactate	27 mmol/L	< 3.3 mmol/L	Markedly elevated
Potassium	6.7 mmol/L	4.0–6.0 mmol/L	Hyperkalemia
Hematocrit	30.3%	39.0%–63.0% (neonate)	Anemia

^a^ Performed on Day of Life 10.

^b^ Performed via purine and pyrimidine panel.

^c^ Performed on Day of Life 11.

On DOL 11, prior to disclosure of the biochemical findings to the family, the infant experienced acute clinical decompensation characterized by oliguria, hypotension, abnormal respiratory pattern, and arrhythmia. He had been stable on room air the previous night but rapidly required escalation to high‐flow nasal cannula. Arterial blood gas revealed severe metabolic acidosis, with elevated lactate and hyperkalemia. The patient progressed to respiratory failure and hypoxia requiring intubation. Due to persistent hypoxia and prior echocardiographic findings, inhaled nitric oxide was initiated. Electrocardiogram showed a wide complex rhythm with prolonged QTc (QTcB 472 ms). Chest and abdominal radiographs were unremarkable. A head ultrasound could not be completed due to the patient's critical condition. Continuous electroencephalogram (EEG) monitoring during this period revealed a markedly abnormal background with excessive discontinuity and multifocal sharp wave activity, particularly in the bilateral temporal and right occipital regions. Although no seizures were captured, the presence of frequent interictal discharges indicated significant epileptogenic potential. Compared to previous recordings, the interburst intervals progressively lengthened, culminating in a suppressed background prior to the patient's terminal event.

Despite maximal vasopressor support (dopamine and epinephrine), target mean arterial pressures were not achieved. Resuscitative efforts included packed red blood cell transfusion, multiple boluses of epinephrine, normal saline, sodium bicarbonate, calcium chloride, insulin‐dextrose, and chest compressions. The family was present at bedside when resuscitation was discontinued, and time of death was declared. Blood cultures obtained during the decompensation remained negative after five days.

**FIGURE 1:**
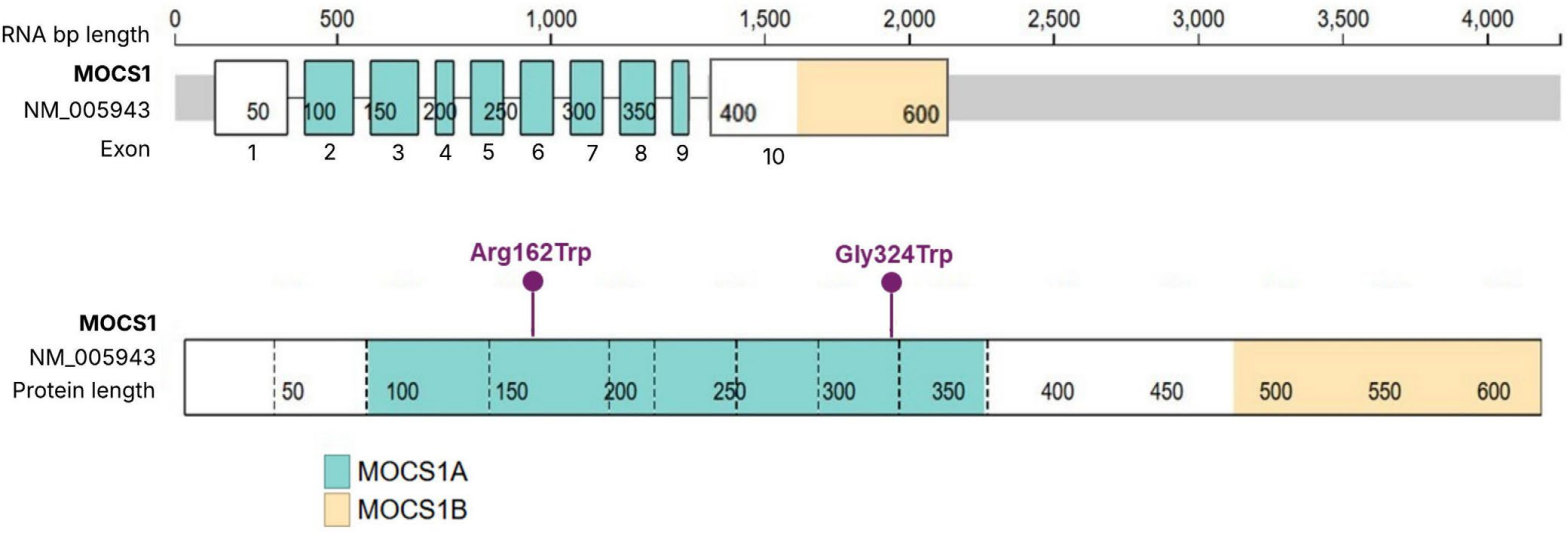
Schematic of *MOCS1* RNA and protein showing two homozygous missense variants identified in the patient: Arg162Trp and Gly324Trp (highlighted in purple). The MOCS1A and MOCS1B domains are indicated in teal and peach, respectively, based on domain annotations from Mayr et al. [13]. The illustration was generated using ProteinPaint with the NM_005943 transcript. Notably, the MANE Select reference transcript for *MOCS1* is NM_001358530, and both variants correspond to the same amino acid positions in this transcript.

Rapid genome sequencing results, reported 8 days postmortem, identified homozygosity for a complex allele in *MOCS1* (NM_005943.5; Figure 1), comprising two likely pathogenic missense variants—c.484C>T (p.R162W) and c.970G>T (p.G324W), located in exons 3 and 7, respectively—both inherited in *cis* from each parent. These findings are consistent with a diagnosis of MoCD type A (MIM: 252150). Additionally, a homozygous variant of uncertain significance in *CUL7* (c.3280C>T, p.R1094C) was identified, associated with 3M syndrome (MIM: 273750), a condition typically characterized by asymmetric intrauterine growth restriction, distinct facial features, and skeletal anomalies—none of which were observed in this patient. A secondary finding included a heterozygous, paternally inherited pathogenic variant in *KCNQ1* (NM_000218.2; c.905C>T, p.A302V), associated with long QT syndrome (MIM: 192500). The family was subsequently counseled in the genetics clinic, including discussion of implications for paternal cardiac monitoring and management.

## 4 Discussion

We present a case of MoCD type A with a rapid decline following a period of relative stability. This case underscores several critical challenges in the field of biochemical genetics.

First, as disease‐modifying therapies for IEMs become more widely available, it is essential to establish protocols that expedite the diagnostic workup for potentially treatable conditions. Even when treatment exists and newborn or prenatal screening is feasible, understanding the natural history of a disorder remains vital for appropriate family counseling and individualized patient management. This is particularly challenging in rare diseases, where data are often limited.

In this case, a biochemical diagnosis of MoCD was achieved within 13 h after specimen collection; however, the result was not available in time to alter the clinical course. For neonates and infants presenting with refractory seizures, early biochemical testing—including urinary S‐sulfocysteine and uric acid—can significantly narrow the differential diagnosis. At our institution, the DUHS Biochemical Genetics Laboratory provides preliminary S‐sulfocysteine results within 24 h for infants under 6 weeks of age. A biochemical profile consistent with MoCD typically includes elevated urinary *S*‐sulfocysteine and low or undetectable uric acid, whereas isolated sulfite oxidase deficiency presents with elevated S‐sulfocysteine and normal uric acid [1]. When this biochemical pattern is observed, treatment initiation may be justified even before molecular confirmation [9]. Urinary xanthine and hypoxanthine levels also help differentiate between MoCD and isolated sulfite oxidase deficiency.

This expedited biochemical protocol enabled a faster diagnostic approach than waiting for molecular results alone, highlighting the importance of integrating both testing modalities; however, despite this rapid workup, the patient's clinical deterioration was unexpectedly swift.

Given the severity of the presentation, we considered whether the homozygous complex allele in *MOCS1*—comprising two likely pathogenic missense variants (c.484C>T, p.R162W and c.970G>T, p.G324W)—may have contributed to the phenotype [7]. The *MOCS1* transcripts can undergo alternative splicing resulting in either one or two open reading frames, ultimately producing either the monocistronic mRNA transcript (creating the MOCS1AB fusion protein, from which the MOCS1B protein is cleaved) or the bicistronic mRNA transcript (creating the MOCS1A and MOCS1B open reading frames, of which only MOCS1A is the functional protein) [3, 12]. The MOCS1A domain is encoded by exons 2 through 9, and the MOCS1B domain is encoded by exon 10 [13]. Our patient's missense variants mapped to exons 3 and 7, and therefore could theoretically impact only the MOCS1A protein (Figure 1); however, given that alternative splicing mechanisms are implicated in *MOCS1*, the impact of the two missense variants identified in the patient is unclear at this time as their effect on enzymatic activity has not been elucidated. The finding of one homozygous likely pathogenic variant is sufficient to explain the patient's disease, but the presence of the additional homozygous variant could act as a genetic modifier to affect disease outcome.

We also considered the potential contribution of the pathogenic *KCNQ1* variant, associated with long QT syndrome and sudden cardiac death [14, 15]. Although the variant was inherited from the father and is also present in the patient's four‐year‐old brother, neither of whom have a history of cardiac issues, its role in the patient's rapid deterioration remains unknown. Nevertheless, the identification of this variant has prompted cardiac monitoring in both the father and patient's brother, which may have long‐term benefits. Variants of uncertain significance in *CUL7* were deemed unrelated, as the phenotype of 3M syndrome does not overlap with this case [16].

The uncertainty of the impact of the patient's *MOCS1* variants highlights that genotype–phenotype correlations are another critical component of natural history. Both variants identified in our patient have been previously reported, including one case with both variants in a compound heterozygous state, though without phasing nor clinical details [17]. A related *MOCS1* variant, p.G324E, has been described as a founder mutation in Israel, with affected individuals experiencing early mortality ranging from 6 to 1549 days [3, 18]. These outcomes are more severe than those reported in broader natural history studies, where median survival ranges from 2.4 to 3.0 years [18]. Notably, the most common causes of death in MoCD are lower respiratory infections and seizures [3].

As more disease‐modifying therapies emerge, early detection and immediate treatment initiation are crucial. Although cPMP was FDA‐approved in 2021, it is often not readily available at centers for many reasons including administration requiring daily intravenous infusion and the rare nature of the disease [8]. It is uncertain whether initiation of treatment would have altered this patient's outcome, but this case highlights the need for improved preparedness. Literature reports show that infants treated within hours of birth, following prenatal diagnosis, have achieved survival beyond 41 months with developmental progress [9, 19]. Prenatal clues, such as a prominent cisterna magna (observed in our case), are beginning to emerge as potential diagnostic indicators [18, 20].

Beyond individual care, MoCD is being considered for inclusion in NBS programs. Elevated xanthine and low uric acid levels can be reliably detected on dried blood spots by DOL 3 [21]. MoCD meets several Wilson and Jungner criteria for NBS: early onset, detectable biomarkers before symptom onset, and availability of effective treatment [22, 23]. This raises broader questions about the scalability of NBS as more treatable IEMs are identified [23]. In the case of our patient, presumably, a NBS result would have identified MoCD sooner than when it was identified in the clinical setting (DOL 10).

Unanswered questions about the severe nature of this case remain. It is noteworthy that while in‐house targeted testing revealed a marked elevation of urinary S‐sulfocysteine analysis, a panel performed by a different laboratory on urine collected the same day showed elevated levels, though lower than the targeted test. This discrepancy may reflect inter‐laboratory variation in sample preparation and/or analytical methods. While plasma uric acid was undetectable, urinary uric acid was detectable at a concentration comparable to that reported in a mild case of *MOCS1* deficiency [24]. Reduced urinary uric acid combined with elevated S‐sulfocysteine has been proposed as a marker of more severe clinical presentation [24]; however, other studies suggest that age at diagnosis, age of treatment, and degree of brain involvement prior to treatment initiation are stronger prognostic indicators than biochemical findings alone [3, 25, 26]. Furthermore, unlike previously published cases of MoCD with elevated urinary hypoxanthine [2], our patient exhibited normal hypoxanthine levels. Thus, the patient presented had overlapping features of both mild and severe cases of MoCD in the literature.

Although the outcome in this case was abrupt and tragic, it offers valuable insights. Most importantly, it reminds us that while rapid biochemical and molecular diagnostics have advanced significantly, there is still room for improvement. This patient's story inspires continued efforts to refine early detection, expand access to treatment, and deepen our understanding of rare metabolic diseases. Moreover, the identification of a pathogenic *KCNQ1* variant in this patient led to cardiac monitoring in asymptomatic family members, illustrating how comprehensive genetic evaluation can facilitate early detection and management of incidental but life‐threatening conditions.

## Author Contributions

**Molly M. Crenshaw:** conception and design, data analysis, data interpretation, and article drafting and revising. **Yasmeen Midgette:** data analysis, data interpretation, and article drafting and revising. **Shruthi Mohan:** data analysis, data interpretation, and article drafting and revising. **Ruhan Wei:** data analysis, data interpretation, and article revising. **Mariele Anneling:** data analysis, data interpretation, and article revising. **Monika Williams:** data analysis, data interpretation, and article revising. **Milap Patel:** data analysis, data interpretation, and article revising. **Benjamin T. Cocanougher:** data analysis, data interpretation, and article revising. **Sarah P. Young:** conception, data analysis, data interpretation, and article revising. **Dmitriy Niyazov:** conception, data analysis, data interpretation, and article revising. **Areeg El‐Gharbawy:** conception, data analysis, data interpretation, and article revision. All of the authors provided agreement for submission. **Ashlee R. Stiles:** conception and design, data analysis, data interpretation, and article drafting and revising.

## Funding

The authors have nothing to report.

## Ethics Statement

An ethics approval was not required for this study.

## Consent

Consent was obtained from the family.

## Conflicts of Interest

The authors declare no conflicts of interest.

## Data Availability

The data that support the findings of this study are available on request from the corresponding author. The data are not publicly available due to privacy or ethical restrictions.
